# Design and Experimental Results of a Three-Dimensional Force Sensor for Shearer Cutting Pick Force Monitoring

**DOI:** 10.3390/s23239521

**Published:** 2023-11-30

**Authors:** Bing Miao, Yunwang Li, Yinan Guo, Xiusong You, Shirong Ge

**Affiliations:** 1 School of Mechanical and Electrical Engineering, China University of Mining and Technology (Beijing), Beijing 100083, China; 2 Key Laboratory of Intelligent Mining Robotics, Ministry of Emergency Management, Beijing 100083, China

**Keywords:** three-dimensional force sensor, digital twinning, cross-coupling

## Abstract

The main focus of this work is the design and development of a three-dimensional force sensor for the cutting pick of a coal mining shearer’s simulated drum. This sensor is capable of simultaneously measuring the magnitude of force along three directions of the cutting pick during the cutting sample process. The three-dimensional force sensor is built based on the strain theory of material mechanics, and reasonable structural design is implemented to improve its sensitivity and reduce inter-axis coupling errors. The strain distribution of the sensor is analyzed using finite element analysis software, and the distribution of the strain gauges is determined based on the analysis results. In addition, a calibration test system is designed for the sensor, and the sensitivity, linearity, and inter-axis coupling errors of the sensor are calibrated and tested using loading experiments in three mutually perpendicular directions. Modal simulation analysis and actual cutting pick testing of the coal mining machine’s simulated drum are conducted to study the dynamic characteristics and functionality of the sensor in practical applications. The experimental results depict sensitivities of 0.748 mV/V, 2.367 mV/V, and 2.83 mV/V for the newly developed sensor, respectively. Furthermore, the cross-sensitivity error was lower than 5.02%. These findings validate that the sensor’s structure satisfies the measurement requirements for pick-cutting forces.

## 1. Introduction

Although new clean energy technologies are currently developing rapidly, fossil energy remains the primary global energy source. In 2022, global energy demand increased by 1% compared to the previous year, with fossil fuels accounting for 82% of the energy supply. China’s total energy consumption reached 5.41 billion metric tons of standard coal in 2022, representing a 2.9% increase from the previous year. Among underground coal mining methods, longwall mining using a shearer is the most commonly employed. The output and productivity of longwall mining depend on the cutting performance of the shearer on the longwall face [1,2]. The cutting load, which represents the working load of the shearer, is a primary criterion in the design of the shearer.

Cutting force pertains to the force applied by the cutting pick of a coal mining shearer onto the coal rock. The measurement of cutting force allows for the determination of whether the coal mining shearer is cutting through coal or rock. Consequently, cutting force serves as a crucial parameter in evaluating the overall effectiveness of the coal mining process [3]. With the establishment of a precise and dependable cutting force measurement system, the mining process of the coal mining shearer can be effectively monitored, ensuring safety and enhancing mining efficiency. Through the measurement of the three-dimensional forces exerted on the cutting pick of the simulated drum on the coal mining shearer, the effects of different geometric shapes, tooth pitch, cutting depth, tooth angle, and rotation speed on the cutting load can be analyzed. A cutting load model is then constructed and extrapolated to encompass the actual working conditions of the coal mining shearer.

To address this concern, researchers from academia and industry have proposed various methodologies for measuring the cutting force of a shearer’s drum pick. Yu [4] and Mishra [5] employed the finite element method (FEM) using LS-DYNA and ABAQUS/Explicit to establish a rotating drum-cutting model. This model aimed to examine the impact of rotational speed and the geometric shape of the pick on the cutting load of the drum. However, in real-world scenarios, the pick experiences dynamically variable loads over time. Modeling the tooth structure with finite element or discrete element analysis involves connecting numerous elements to form a discrete object or structure, resulting in an approximate numerical simulation. Liu et al. [6] proposed an effective numerical and experimental method for studying the interaction between multiple picks and coal rock and selecting the best pick layout parameters. The three forces exerted by the conical picks are collected by the load sensor in real time and transmitted to the dynamic strain indicator simultaneously. Huang et al. [7] and Qiao et al. [8] proposed a similar experimental test system consisting of the compressive machine, the pick holder, the conical pick, the rock box, the rock sample, and a computer. The compressive machine can provide maximum force and can record the variation of the force and the displacement. However, they are all measured using a single-dimensional force transducer. Zhang et al. [9] proposed that a welded strain gauge sensor should be used to test the cutting load of the picks, and a strain gauge rosette sensor should be used to measure the torsional stress. In the experiment, a welded strain gauge sensor was connected to an adjacent wireless strain acquisition module through a wire, and a total of six picks were selected for the cutting load test. However, the sensitivity and error of the sensor are not calibrated and measured. Liu [10] developed an automated rotating cutting simulation test rig to investigate the effects of tooth geometric shape, tooth line spacing, cutting depth, tooth inclination angle, and rotational speed on cutting loads. Nevertheless, due to the substantial size of the three-dimensional force sensor, experiments can only be conducted with a single tooth, thus failing to consider the influencing factors of multiple picks.

Both of the aforementioned methods fail to fully capture the cutting force exerted by the cutting tooth in the shearer’s actual operational conditions. Therefore, there is an urgent need to develop three-axis force sensors for cutting picks that can be directly mounted on the shearer’s actual or simulated drum blades. These sensors enable the monitoring of the three-dimensional force distribution during the rotational cutting process of the drum. The three-dimensional force sensor for cutting picks should meet the following requirements:

- Compact structure for easy installation on drum blades and simultaneous operation with other picks;
- Reduction of input–output non-linearity in the three-dimensional force sensor system;
- Minimization of coupling interference between dimensions.

Currently, two methods are employed to mitigate inter-dimensional coupling: structural decoupling and algorithmic decoupling. In the realm of structural decoupling, scholars have explored the crossbeam structure [11,12], the Stewart structure [13,14,15], and the spoke-type structure [16]. Sina [17] employed three sets of strain gauges positioned on three cylindrical probes to create a three-dimensional stress sensor. However, this approach featured a complex strain gauge layout that led to notable inter-dimensional coupling. Hu [18] developed an enhanced crossbeam elastic structure with strain gauges attached to the front and sides of the elastic beam. Although this configuration offered a simpler layout, it still exhibited significant inter-dimensional coupling. Building upon these findings, Xiong [19] introduced a layered perception elastic structure, incorporating a torque measurement layer, an absorption layer, and a force measurement layer to minimize coupling interference between force and torque. However, this design still experienced coupling interference between the axial force in the force measurement layer and a pair of horizontally orthogonal forces. These aforementioned structures suffer from inter-dimensional coupling interference and require precise manufacturing and installation.

This study builds upon the mentioned structures to enhance force measurement performance. A novel and compact sensor for measuring three-dimensional cutting force in cutting picks is proposed.

Firstly, we determine the dimensions of the 3D force sensor based on the structure of the simulated drum. Secondly, we calculate the measuring range of the 3D force sensor based on the force formula when the simulated drum is cutting coal and rock samples. Finally, we design the structure of the 3D pick force sensor (corresponding to Section 2.1, Section 2.2 and Section 2.3 in this paper).

The layout of the strain gauges is determined based on the finite element method (FEM) simulation results, which enable mechanical decoupling of the three-dimensional cutting force. Additionally, the modal parameters of the sensor are calculated using FEM simulation to verify its dynamic performance (corresponding to Section 2.4 in this paper).

A calibration and testing apparatus, along with a data acquisition and analysis system, is designed specifically for the three-dimensional force sensor. The sensor’s performance is evaluated through static calibration of the three-dimensional force (corresponding to Section 3 in this paper).

**Figure 1:**
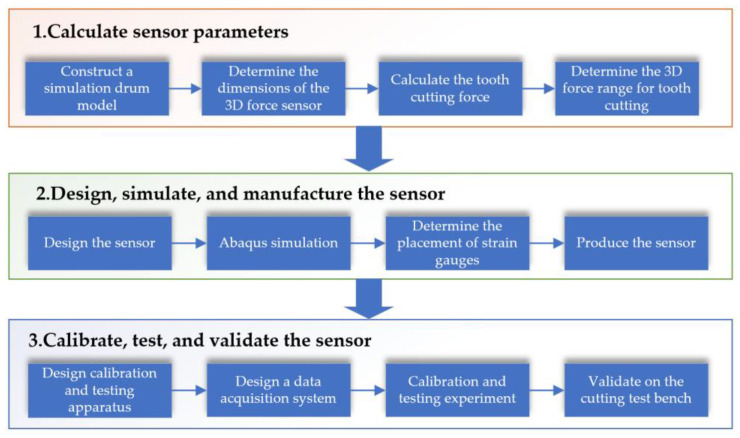
Three-dimensional force sensor design process diagram.

By processing the data from the sensor, the sensitivity and cross-sensitivity error of the sensor are calculated and analyzed. Finally, the designed sensor is mounted on a simulated drum of a shearer, and cutting tests are carried out on a CNC coal rock cutting test bench to obtain three-dimensional force data during the cutting process (corresponding to Section 4 in this paper). The design process diagram of the sensor is shown in the Figure 1.

## 2. Design and Sensing Method of Three-Dimensional Force Structure for Simulated Drum

### 2.1. Structure and Size Design of Three-Dimensional Force Sensor

**Figure 2:**
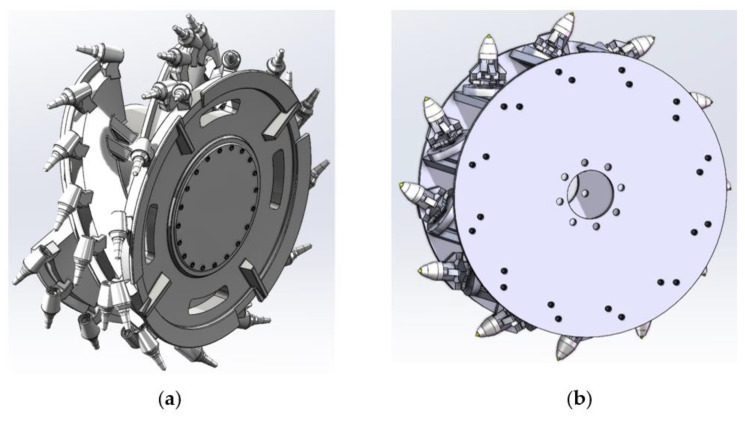
(**a**) Coal mining shearer drum; (**b**) simulated drum.

The coal mining shearer features a significant number of cutting picks on its cutting drum, posing challenges in measuring the instantaneous forces exerted on each tooth during the cutting process. To tackle this issue, a simulated drum for the coal mining machine was designed, taking into consideration the actual working conditions of the drum and the layout characteristics of the cutting pick, as shown in Figure 2.

In adherence to similarity criteria, the size of the simulated drum was reduced, enabling the simulation of parameters such as the number of cutting-edge lines, helix angle, and installation angle of the drum blades, as indicated in Table 1.

**Figure 3:**
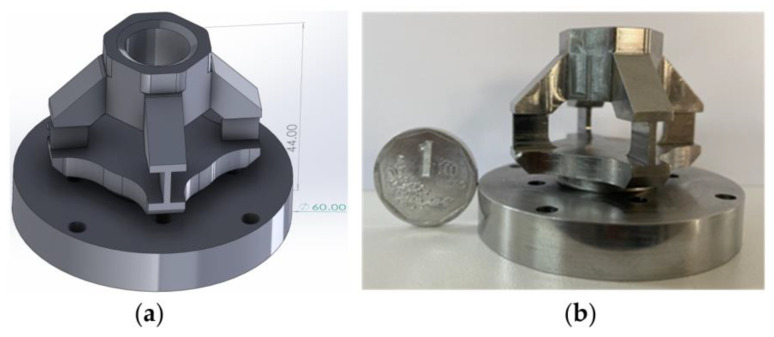
(**a**) Three-dimensional force sensor model; (**b**) the actual three-dimensional force sensor.

Consequently, the dimensions of the toothed three-dimensional force sensor should conform to the size specifications of the simulated drum of the coal mining machine, as depicted in Figure 3. The three-dimensional force sensor has a height of 44 mm and a bottom diameter of 60 mm.

To overcome the issues of positional sensitivity, low accuracy, and weak resistance to bias load in normal stress measurement, the three-dimensional force sensor adopts the principles of shear stress sensing or shear beam sensing. The elastic body of the sensor is designed as an integrated structure and processed as a whole. The base is a circular structure, with a cylindrical ring at the center for mounting the gear tooth, while the four pillar-shaped elastic bodies connect the base to the cylindrical ring. The base is fixed to the drum blade using bolts.

### 2.2. Calculating the Measuring Range of the Three-Dimensional Force Sensor

**Figure 4:**
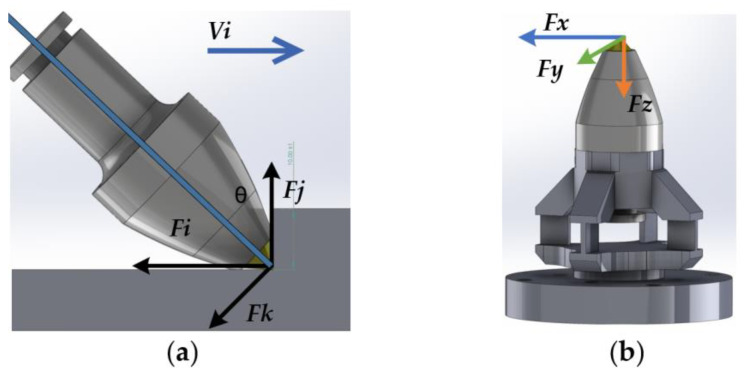
(**a**) Single cutter cutting force; (**b**) three-axis definition of three-dimensional force sensor.

The force situation of a single cutting tooth on the shearer’s drum is shown in Figure 4. Based on the mechanical parameters of the simulated drum of the shearer and the properties of the coal and rock specimens to be cut, the design range of the three-dimensional force sensor was calculated and determined.

The cutting tooth is subjected to three directions of load:

- F_i_ represents the cutting load, which is opposite to the cutting speed Vi of the cutting tooth;
- F_j_ represents the cutting normal load, which is perpendicular to the direction of the cutting speed of the cutting tooth;
- F_k_ represents the cutting lateral load, which is perpendicular to the plane formed by the cutting load F_i_ and the cutting normal load F_j_, pointing towards the goaf.

The cutting tooth is mainly subjected to the above three directions of load. Based on the theory of rock fracture mechanics, Li et al. [20,21] analyzed the fracture mechanics mechanism of pick-type cutting teeth for rock breaking. He established a theoretical model for calculating the peak cutting force of the pick-type cutting tooth based on rock fracture cutting. The calculation formula for the cutting load F_i_ is as follows:

(1) $$xF_{i}\left(t\right)=f_{\mathrm{rel}}\cdot K_{y}\cdot \mathrm{Norm}\left({\mathrm{PCR}}_{\mathrm{ci}}/r_{1},{\mathrm{PCR}}_{\mathrm{ci}}/r_{2}\right)$$

(2) $$\left\{\begin{array}{l} f_{\mathrm{rel}}=0.399+0.667\cdot s/d\\ K_{y}=c+\left(h/H-0.1\right)/\left(h/H+1\right)\\ {\mathrm{PCR}}_{\mathrm{ci}}=\frac{\lambda^{\frac{5}{6}}K_{\mathrm{IC}}d^{\frac{5}{3}}}{3\times \sqrt[{6}]{\frac{\pi^{2}}{12\cos\beta_{1}}\left[\frac{1}{3}{\left(\frac{\sin\alpha_{1}}{\cos\beta_{1}}-1\right)}^{2}+1\right]}} \end{array}\right.$$

In the formula, f_rel_ is the cutting load coefficient affected by intercept; K_y_ is the tension coefficient on the coal wall surface; Parameters of coal rock specimens as shown in Table 2; α_1_ is the semi-tip angle of the conical pick; β_1_ is the rake angle; and PCR_ci_ represents the peak cutting load on the cutting pick.

F_k_ represents the cutting lateral load, which can be calculated according to the formula proposed by Liu [22]:

(3) $$F_{k}\left(t\right)=F_{i}\left(t\right)\cdot \left(\frac{a_{1}}{a_{2}+0.1\times d}+a_{3}\right)\cdot \frac{d}{s}$$

when the cutting picks are arranged in order, a_1_ = 1.4 × 10^−3^, a_2_ = 0.3 × 10^−3^, and a_3_ = 0.15. During coal cutting, the normal load F_j_ acting on the cutting pick can be obtained using the following equation:

(4) $$F_{j}\left(t\right)=F_{i}\left(t\right)\cdot \cot\theta$$

Based on the parameters of the simulated drum of the coal mining machine and the coal rock specimen, the following calculations are obtained. The cutting load range of the cutting pick Fi is 0–4.5 KN. Then, we convert the coordinate system from F_i_, F_j_, F_k_ to F_x_, F_y_, F_z_; here, F_k_ corresponds to F_y_, and θ is the angle between F_j_ and F_z_.

(5) $$\left[\begin{array}{c} F_{x}\\ F_{y}\\ F_{z} \end{array}\right]=\left[\begin{array}{ccc} \cos\theta & 0 & -\sin\theta \\ 0 & 1 & 0\\ \sin\theta & 0 & \cos\theta \end{array}\right]*\left[\begin{array}{c} F_{i}\\ F_{k}\\ F_{j} \end{array}\right]$$

Therefore, the range of the designed three-dimensional force sensor is set as follows: F_z_ = 5 KN; F_x_ = F_y_ = 2 KN.

### 2.3. Method and Principle of Three-Dimensional Force Sensing

The basic principle of resistive strain gauge detection is that external force causes a slight elastic deformation on the force-sensitive element. This deformation is converted into a resistance change that can be measured by the circuit using the strain gauges and then transformed into voltage or current output via a bridge circuit. Ultimately, a decoupling algorithm is employed to calculate the measurement result of the external force. By replacing the resistances on the arms of the bridge with different numbers and in different ways using strain gauges that satisfy certain relationships with the bridge arms, measurement schemes such as 1/4 bridge, half-bridge, and full-bridge can be formed. All three schemes can be used to measure strain, but in order to improve the sensitivity of the sensor and reduce the influence of environmental temperature, it is advisable to adopt a full-bridge detection circuit that features temperature compensation and non-linear error correction.

(6) $$\left\{\begin{array}{l} U_{0}=\frac{U_{\mathrm{vcc}}}{4}\left(\begin{array}{l} \frac{\Delta R_{1}}{R_{1}}-\frac{\Delta R_{2}}{R_{2}}+\frac{\Delta R_{4}}{R_{4}}-\frac{\Delta R_{3}}{R_{3}} \end{array}\right)\\ R_{1}=R_{2}=R_{3}=R_{4}\\ \Delta R_{1}=-\Delta R_{2}=-\Delta R_{3}=\Delta R_{4} \end{array}\right.$$

**Figure 5:**
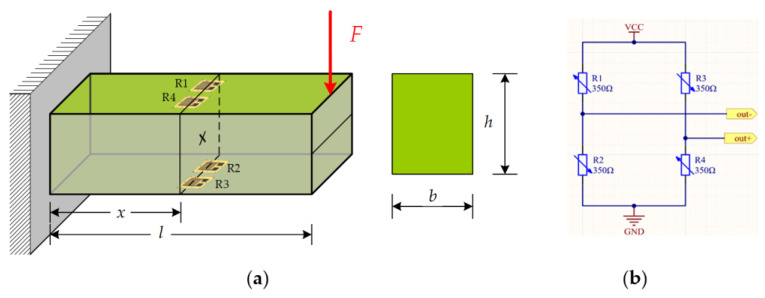
(**a**) Cantilever beam with strain gauge; (**b**) full-bridge detection schematic.

In material mechanics, the strain at the observation section X on the cantilever beam caused by the force applied at the free end of the beam is demonstrated using the equation:

(7) $$\varepsilon =\frac{\left(l-x\right)\mathrm{hF}}{2\mathrm{EI}}=\frac{6F\left(l-x\right)}{{\mathrm{Ebh}}^{3}}$$

where E is the elastic modulus of the cantilever beam; ε is the strain at the observation point; F is the force applied at the free end of the cantilever beam; x is the distance from the observation point to the fixed end of the cantilever beam; I is the moment of inertia of the section at x; the symbols *l*, h and b are the length, height and thickness of the cantilever beam, as shown in Figure 5.

The cross-coupling effect in a three-dimensional force sensor refers to the phenomenon where forces or moments in one direction can affect the measurements in other orthogonal directions. Cross-coupling can affect the accuracy and reliability of the force measurements and needs to be carefully taken into account during calibration and data processing.

(8) $$\left[\begin{array}{c} F_{x}\\ F_{y}\\ F_{z} \end{array}\right]=\left[\begin{array}{ccc} K_{\mathrm{xx}} & K_{\mathrm{yx}} & K_{\mathrm{zx}}\\ K_{\mathrm{xy}} & K_{\mathrm{yy}} & K_{\mathrm{zy}}\\ K_{\mathrm{xz}} & K_{\mathrm{yz}} & K_{\mathrm{zz}} \end{array}\right]*\left[\begin{array}{c} \Delta \varepsilon_{x}\\ \Delta \varepsilon_{y}\\ \Delta \varepsilon_{z} \end{array}\right]$$

where Fx, Fy, and Fz represent the forces in the X-, Y-, and Z-directions, respectively, and ∆ε_x_, ∆ε_y_, and ∆ε_z_ represent the output values of the strain sensors parallel to the X-, Y-, and Z-directions, respectively. Kij represents the coefficient of the relationship between the force in the i-direction and the output of the strain gauges parallel to the j-direction, where i can be X, Y, or Z, and j can be X, Y, or Z.

### 2.4. Simulation Analysis

In order to conduct strain analysis, the finite element software Abaqus/CAE 2022 was used to simulate the sensor elastomer. Abaqus, a powerful finite element analysis (FEA) software, is widely employed in the fields of engineering and science. It effectively handles the irregular shape of 3D force sensors through the utilization of its adaptive mesh function. This functionality automatically refines or coarsens the mesh based on specified requirements, leading to efficient utilization of computational resources and the generation of more precise results. Abaqus offers a broad spectrum of material models, enabling users to accurately simulate complex material behaviors. It is capable of performing not only static strain analysis on sensors but also of facilitating additional simulations such as gear cutting of coal and rock. To improve the analysis accuracy, the chamfers of the sensor, which could potentially affect the results of finite element analysis, were simplified. The hexahedral meshing technique was employed with a mesh size of 1 mm. A 316 L stainless steel was selected as the material for the elastomer, and its structural parameters are detailed in Table 3.

**Figure 6:**
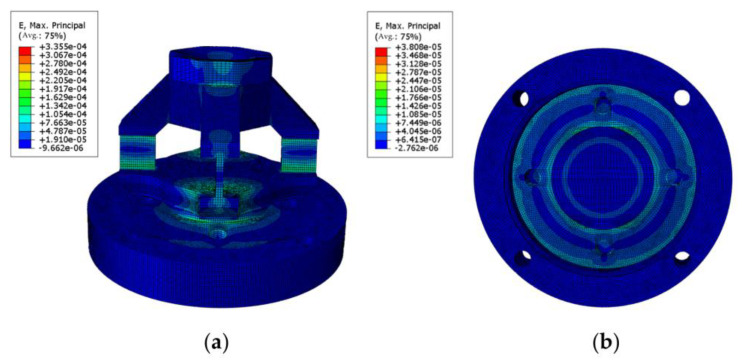
(**a**) FEM analysis of force on *X*- or *Y*-axes; (**b**) FEM analysis of force on *Z*-axes.

According to the actual installation requirements of the sensor elastomer, a surface constraint was applied to the bottom surface of the sensor elastomer. The picks are made of high hardness and wear-resistant alloy steel. During the cutting process, the picks are embedded into the coal and rock to be cut, and the cutting force is transmitted to the top of the sensor through the pick handle. In the FEM analysis, the deformation of the pick and the top of the sensor is ignored, thus loading the force onto the upper surface of the sensor. Three concentrated forces, Fx = 2 KN, Fy = 2 KN, and Fz = 5 KN, were applied at the central point of the upper surface of the elastomer in three different directions. Figure 6 illustrates the strain distribution of the sensor elastomer under the influence of these three applied forces.

**Figure 7:**
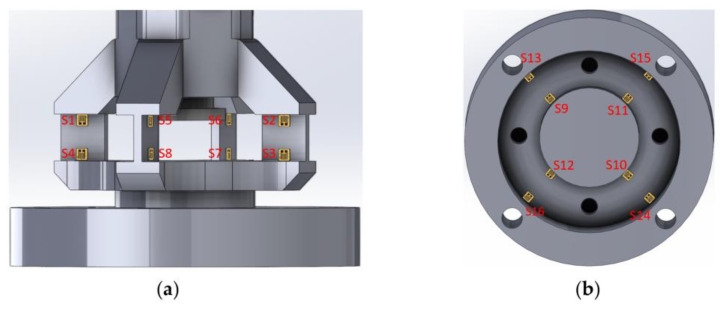
(**a**) Position of strain gauge on *X*- or *Y*-axes; (**b**) position of strain gauge on *Z*-axes.

Based on the FEM analysis, the following conclusions can be drawn. When a force is applied in the X-direction, the elasticity of the material undergoes the maximum strain, with the maximum strain being ε = 3.355 × 10^−4^. It can be inferred from ε × E = 70.79 MPa that the maximum strain of the material is significantly lower than the yield strength of the 316 L stainless steel, which is 170 MPa. This indicates that a sensor with an elastic structure is safe to use. Based on the simulation results, the mounting position of the strain gauge can be determined, as shown in Figure 7.

The X-direction force mainly affects the strain gauge S1–4, and the Y-direction strain gauge S5–8; the Z-direction inner ring strain gauge S9–12 and the outer ring strain gauge S13–16 have little influence. The strain values of the strain gauges S1–2 and S3–4 are almost equal, but the signs are opposite, indicating that the strain gauge S1–2 is stretched and the strain gauge S3–4 is compressed under the action of the X-direction force. Therefore, the strain under the action of load Fx can be obtained by calculating the strain difference between strain gauges S1–2 and S3–4.

The force in the Y-direction primarily affects strain gauges S5–8, strain gauges S1–4 in the X-direction, as well as the inner-ring strain gauges S9–12 and the outer-ring strain gauges S13–16 in the Z-direction have little influence. The strain values of S5–6 and S7–8 are almost equal but with opposite signs, indicating that S5–6 experiences compression and S7–8 experiences tension under the force in the Y-direction. Therefore, the strain under load Fy can be calculated by obtaining the difference in strain between S5–6 and S7–8.

The force in the Z-direction has a significant impact on strain gauges S9–16, with equal strain values and the same sign for the inner-ring strain gauges S9–12 and the outer-ring strain gauges S13–16. This suggests that S9–12 experiences compression and S13–16 experiences tension under the force in the Z-direction. Therefore, the strain under the load Fz can be calculated by obtaining the difference in strain between S9–12 and S13–16.

The X-direction load measurement circuit of the three-dimensional force sensor adopts a four-arm differential full-bridge circuit. RS1 to RS4 respectively represent the initial resistance values of strain gauges S1 to S4, and Us represents the power supply voltage of the bridge. Therefore, the calculation formula for the output voltage of this circuit is as follows:

(9) $$\mathrm{Ux}=\frac{R_{S1}R_{S2}-R_{S3}R_{S4}}{\left(R_{S1}+R_{S4}\right)\left(R_{S2}+R_{S3}\right)}U_{s}$$

Assuming that the selected strain gauges have the same specifications and their sensitivity coefficient is K_R_, according to Formula (9), we can obtain:

(10) $$\mathrm{Ux}=\frac{K_{R}U_{s}}{4}\frac{\varepsilon_{S1}+\varepsilon_{S2}-\varepsilon_{S3}-\varepsilon_{S4}}{1+\frac{K_{R}}{2}\left(\varepsilon_{S1}+\varepsilon_{S2}+\varepsilon_{S3}+\varepsilon_{S4}\right)}$$

In the formula, ε_S1_ to ε_S4_ respectively represent the strain values of strain gauges S1 to S4. In addition, based on force analysis, we can derive the following formula:

(11) $$\varepsilon_{S1}=\varepsilon_{S2}=-\varepsilon_{S3}=-\varepsilon_{S4}=\varepsilon_{\mathrm{Sx}}$$

In this formula, ε_Sx_ represents the strain values of strain gauges S1, S2, S3, and S4, where the values are positive when under tension and negative when under compression. Based on the above conditions, we can deduce the following result:

(12) $$\mathrm{Ux}=U_{s}K_{R}\varepsilon_{\mathrm{Sx}}$$

Similarly, the *Y*-axis load measurement circuit of the three-dimensional force sensor is a four-arm differential full-bridge circuit, therefore, we can obtain:

(13) $$\left\{\begin{array}{c} \mathrm{Uy}=\frac{R_{S5}R_{S6}-R_{S7}R_{S8}}{\left(R_{S5}+R_{S8}\right)\left(R_{S6}+R_{S7}\right)}U_{s}\\ \mathrm{Uy}=\frac{K_{R}U_{s}}{4}\frac{\varepsilon_{S5}+\varepsilon_{S6}-\varepsilon_{S7}-\varepsilon_{S8}}{1+\frac{K_{R}}{2}\left(\varepsilon_{S5}+\varepsilon_{S6}+\varepsilon_{S7}+\varepsilon_{S8}\right)}\\ \varepsilon_{S5}=\varepsilon_{S6}=-\varepsilon_{S7}=-\varepsilon_{S8}=\varepsilon_{\mathrm{Sy}}\\ \mathrm{Uy}=U_{s}K_{R}\varepsilon_{\mathrm{Sy}} \end{array}\right.$$

In this formula, RS5 to RS8 respectively represent the initial resistance values of strain gauges S5 to S8; Us represents the power supply voltage of the bridge; the strain gauge sensitivity coefficient is K_R_; εS5 to εS8 represent the strain values of strain gauges S5 to S8; and ε_Sy_ represents the strain values of strain gauges S5, S6, S7, and S8, where the values are positive when under tension and negative when under compression.

In order to reduce the coupling interference of the *X*-axis and *Y*-axis to the *Z*-axis in the three-dimensional force sensor, an eight-resistance four-arm differential full-bridge circuit is adopted for the *Z*-axis. Among them, RS9 to RS16 respectively represent the initial resistance values of strain gauges S9 to S16, and Us represents the power supply voltage of the bridge. Therefore, the following formula can be obtained:

(14) $$\left\{\begin{array}{c} \begin{array}{c} \mathrm{Uz}=\frac{\left(R_{S9}+R_{10}\right)\left(R_{11}+R_{12}\right)-\left(R_{S13}+R_{S14}\right)\left(R_{S15}+R_{S16}\right)}{\left(R_{S9}+R_{S10}+R_{S15}+R_{S16}\right)\left(R_{S11}+R_{S12}+R_{S13}+R_{S14}\right)}U_{s}\\ \mathrm{Uz}=\frac{K_{R}U_{s}}{8}\frac{\varepsilon_{S9}+\varepsilon_{S10}+\varepsilon_{S11}+\varepsilon_{S12}-\varepsilon_{S13}-\varepsilon_{S14}-\varepsilon_{S15}-\varepsilon_{S16}}{1+\frac{K_{R}}{2}\left(\varepsilon_{S9}+\varepsilon_{S10}+\varepsilon_{S11}+\varepsilon_{S12}+\varepsilon_{S13}+\varepsilon_{S14}+\varepsilon_{S15}+\varepsilon_{S16}\right)}\\ \varepsilon_{S9,10,11,12}=-\varepsilon_{S13,14,15,16}=\varepsilon_{\mathrm{Sz}}\\ \mathrm{Uz}=U_{s}K_{R}\varepsilon_{\mathrm{Sz}} \end{array} \end{array}\right.$$

**Figure 8:**
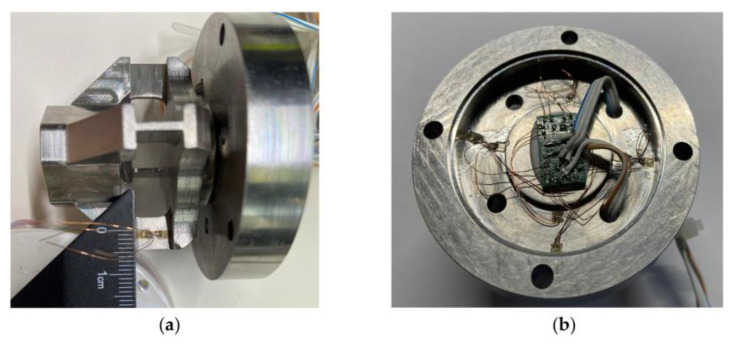
(**a**) Mounting of strain gauges on *X-* or *Y*-axis; (**b**) mounting of strain gauges on *Z*-axis.

In this formula, the strain gauge sensitivity coefficient is K_R_, and εS9 to εS16 represent the strain values of strain gauges S9 to S16, where the values are positive when under tension and negative when under compression. Figure 8 shows the mounting of the strain gauge of the actual sensor.

In summary, by designing the measurement position of strain gauges and the bridge configuration, the output voltage of the bridge circuit can exhibit a specific linear relationship with the measured strain of the strain gauges, thereby achieving the goal of load measurement.

During the cutting process of the coal mining machine drum, the magnitude and direction of the cutting force constantly change, so the dynamic characteristics of the sensor need to be considered. The vibration mode is an inherent characteristic of the sensor device, which can be obtained through experiments or simulation to determine its natural frequency.

**Figure 9:**
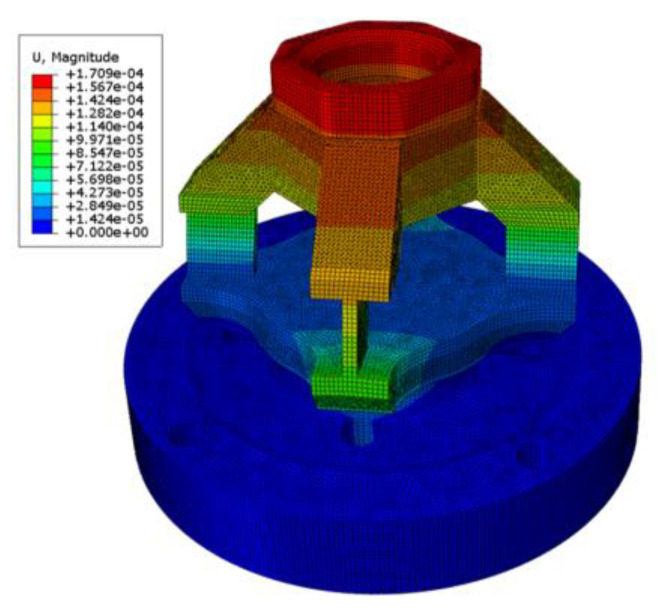
FEM analysis of Modal 1.

To ensure the stability of the measurement process, the natural frequency of the sensor device must be greater than four times the drum vibration frequency (f1) and the tooth force frequency (f2). Among them, the drum vibration frequency (f1) is related to the drum speed. Assuming the working speed of the coal mining machine drum is 60 r/min, the drum vibration frequency f1 is 1 Hz. The results of the modal analysis simulations are shown in Figure 9 and Table 4.

Through experimental verification, the fluctuation period of the interaction between the tooth and the rock during the cutting process can be divided into four stages [21]: Stage I—elastic deformation stage; Stage II—plastic deformation stage; Stage III—formation of main cracks stage; and Stage IV—crack propagation stage. The experimental results indicate that f2 is less than 10 Hz. In the field of engineering, such as finite element analysis, numerical methods can be used to calculate the modal shapes of structures. These shapes are obtained by solving the Eigenvalue problem of the structure, where the mass and stiffness matrices of the structure are used to describe the dynamic characteristics of the system. Generally, higher modal orders correspond to higher frequencies. The first five modal orders are listed. The dynamic simulation evaluation shows that the sensor has a first-order natural frequency of approximately 1.575 kHz, which meets the requirements for operational conditions.

## 3. Design of Calibration Test System

### 3.1. Design of Experimental Apparatus

**Figure 10:**
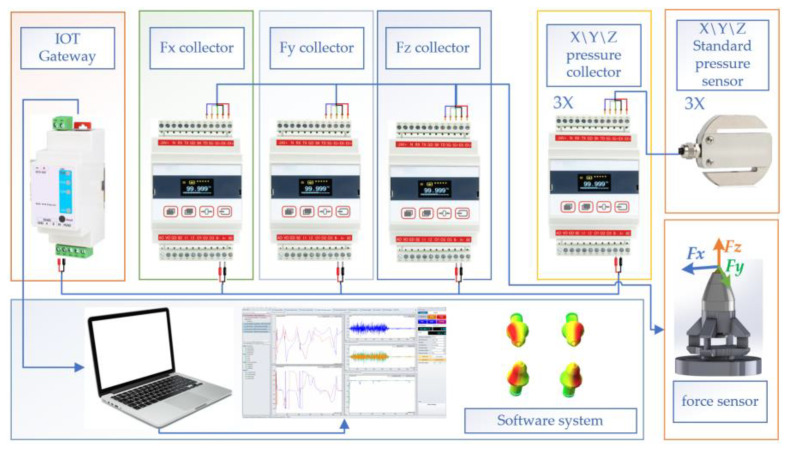
Structure of three-dimensional force testing and calibration system.

The structure of three-dimensional force testing and calibration system are shown in Figure 10.

**Figure 11:**
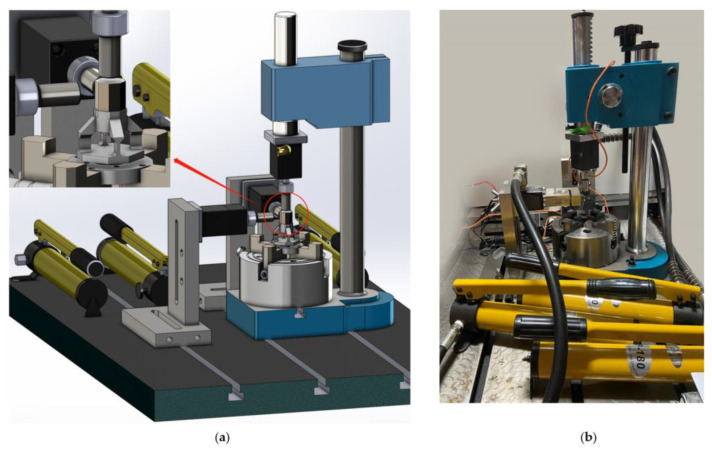
(**a**) Sensor loading device design; (**b**) preparation of sensor loading device.

**Figure 12:**
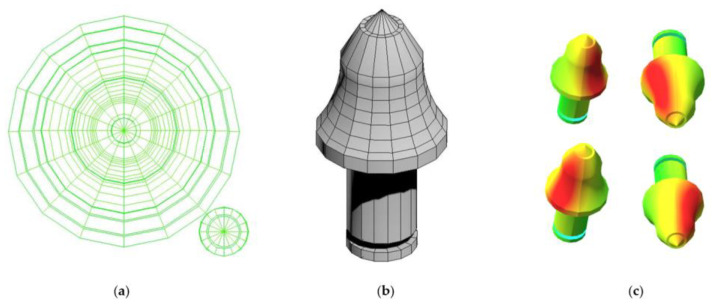
(**a**) Expanded UV grid; (**b**) UV mapping; (**c**) heat map rendering.

1. Sensor loading device: includes T-slot worktable, sensor fixed chuck, mini hydraulic cylinder in *XYZ*-axis direction, and manual hydraulic pump, used for quantitative pressure loading on the three-dimensional force sensor, as shown in Figure 11.

2. Standard pressure sensor and pressure collector are used for real-time measurement of applied pressure, and its parameters are detailed in Table 5.

3. Fx, Fy, and Fz data collector, used for measuring the pressure data of the X, Y, and Z-channels of the three-dimensional force sensor, and its structural parameters are detailed in Table 6.

4. IoT data acquisition gateway: the IoT data acquisition gateway collects the data from the Fx, Fy, and Fz data collector using the Modbus-RTU protocol and converts the collected signal protocol into TCP/IP protocol for uploading to the PC-based digital twin system.
5. PC-based digital twin system: construct a digital twin model of the pick force, adjust the UV mapping of the gear tooth in 3DS Max, and import the created model into Unity 3D, as shown in Figure 12.

On the PC side, use Node.js to build a WebSocket service. Node.js is a server-side JavaScript runtime that is based on the Google Chrome V8 engine and can execute JavaScript code. Node.js itself supports TCP/IP and HTTP protocols and building a WebSocket protocol using Node.js requires additional development using the core HTTP Server library provided by Node.js. This article selects the WS library for building, which can be downloaded and installed directly using NPM.

**Figure 13:**
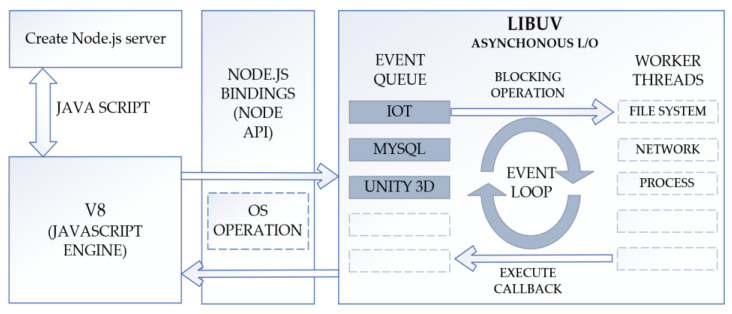
Node-based back-end acquisition system.

Figure 13 illustrates the communication process between the browser and the server using WS. The entire back-end program is responsible for receiving the three-dimensional force sensor data collected by the IoT data acquisition gateway and pushing the data to Unity3D while storing it in the MySQL database.

**Figure 14:**
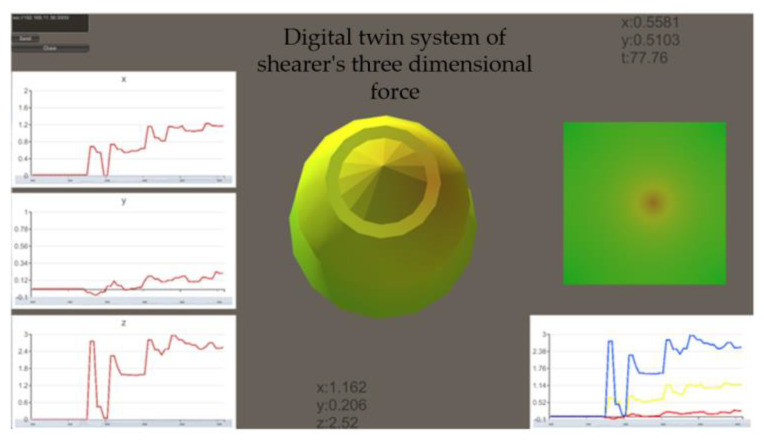
Digital twinning system with three-dimensional force on pick.

Write a C# script file in Unity3D to dynamically update and display the force status of the gear tooth model in the form of a heat map based on the values of the three-dimensional force sensor, as shown in Figure 14.

### 3.2. Sensor Calibration Design

**Figure 15:**
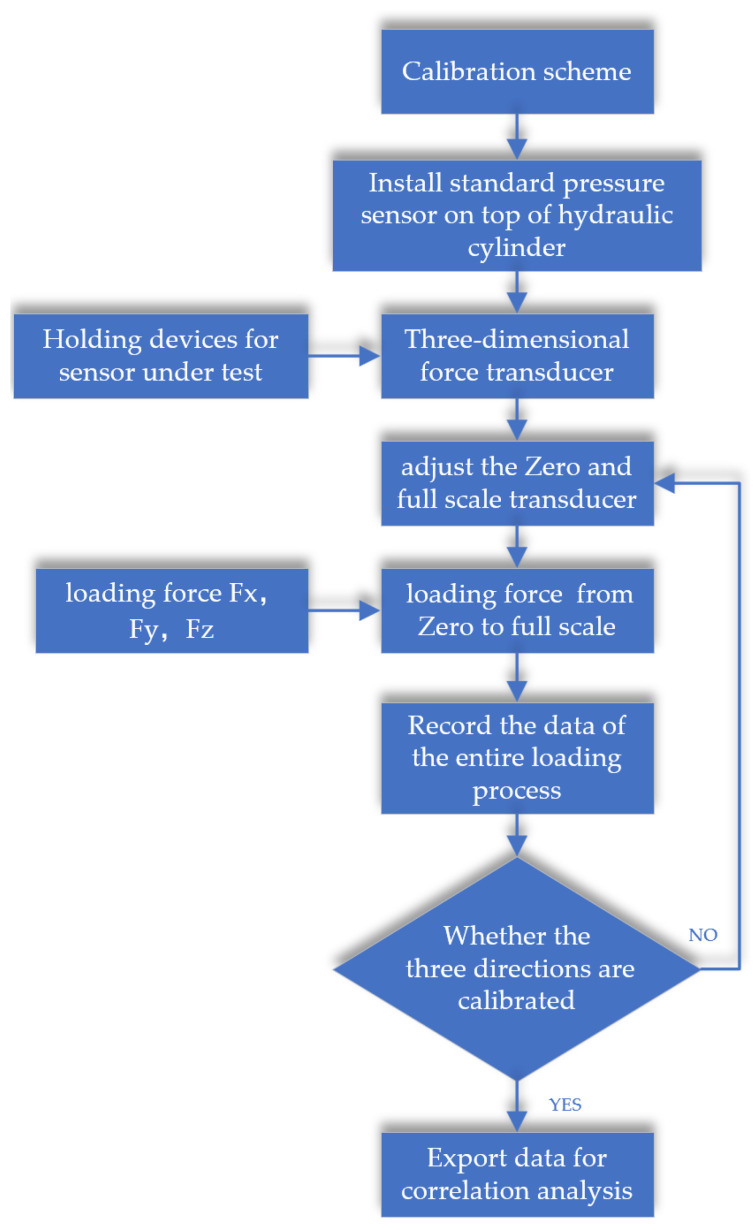
Sensor calibration analysis.

After the analysis mentioned above, after the assembly of the three-dimensional force measuring device, static calibration tests are required to determine the specific relationship between voltage and load. Assuming that the three-dimensional force measuring device’s matrix of three-axis load measurements is represented as F_m×3_ and the matrix of voltage output from the measurement circuit is represented as U_m×3_, they can satisfy the following equation:

(15) $$F_{m\times 3}=U_{m\times 3}K_{3\times 3}$$

where F_m×3_ represents the matrix of three-axis load measurements; U_m×3_ represents the matrix of three-axis voltage output; and K_3×3_ represents the calibration coefficient matrix, which indicates the numerical relationship between the measurement values of the three-dimensional force measuring device and the voltage output from the measurement circuit. Here, m represents the number of data value sequences, representing the data sampling scale. The calibration coefficient matrix is an important guarantee for the accuracy of the three-dimensional force measuring device. It needs to be accurately determined as much as possible through calibration tests and the study of the coupling relationship between load measurements in different directions. The calibration process for the chosen three-dimensional force measuring device is shown in Figure 15.

Fix the three-dimensional force sensor onto the chuck and manually operate the hydraulic pump to apply loads in three directions according to the calibration sequence. Use a standard pressure sensor and pressure collector to obtain the values of the applied loads. Instantly collect the measurement signal values of the three-dimensional force sensor using the Fx, Fy, and Fz data collectors, and communicate with the computer through the IOT data acquisition gateway to achieve data acquisition, data processing, and calibration coefficient settings.

## 4. Test Results and Data Analysis

### 4.1. Sensor Sensitivity Analysis

We installed the test load cell in the tooth installation position of the three-dimensional force sensor and applied a three-directional concentrated force: Fx = Fy = 2 KN and Fz = 5 KN. We measured the power supply voltage of the bridge, the initial offset voltage of the bridge, and the output voltage of the bridge at full load and calculated the initial sensitivity of the corresponding channel. The strain output results are shown in Table 7. We set the range and sensitivity coefficient of the three-dimensional force sensor acquisition instrument according to the measurement results.

**Figure 16:**
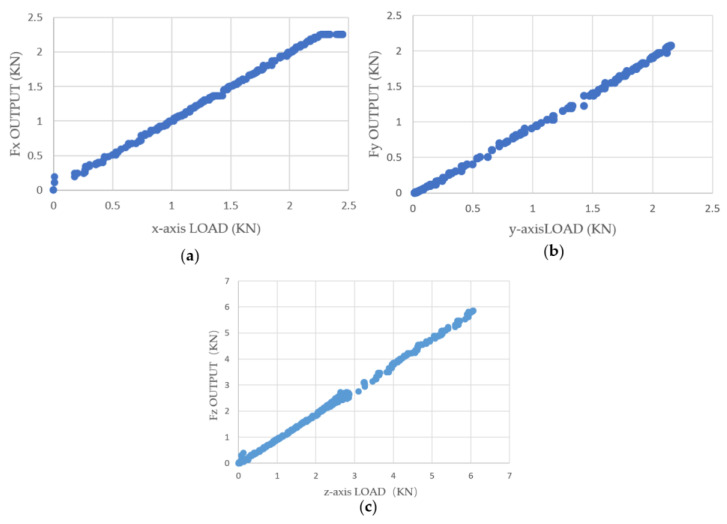
Sensitivity curve: (**a**) *x*-axis direction; (**b**) *y*-axis direction; (**c**) *z*-axis direction.

After setting the range and sensitivity coefficient of the three-dimensional force sensor acquisition instrument, single-axis unidirectional calibration tests were conducted using the three-dimensional force testing device on the *X*-, *Y*-, and *Z*-axes. Graphs were plotted based on the experimental data to illustrate the trends and fitting equations of the input load and output measurements, as shown in Figure 16.

The data in Table 8 and Table 9 are the linear regression results of the sensor under the initial sensitivity setting. The slope in the table represents the fitted slope, the intercept in the table represents the initial intercept, and it also includes the statistically derived Pearson’s r and R^2^.

During single-axis loading, the measurement circuit of the loading axis exhibits good linearity. If we ignore the inter-axis coupling effect and assume that the voltage output of the measurement circuit is only linearly related to the load in its own axial direction, then the calibration coefficient matrix Kss will be a diagonal matrix. The values of the calibration matrix can be obtained by taking the slope of the trend line in the graph.

(16) $$K_{\mathrm{ss}}=\left[\begin{array}{ccc} 0.9493 & 0 & 0\\ 0 & 0.9692 & 0\\ 0 & 0 & 0.9893 \end{array}\right]$$

### 4.2. Sensor Cross-Coupling Effect Analysis

The causes of inter-axis coupling effects in multi-dimensional sensors can generally be divided into structural coupling and error coupling:

- Structural coupling is formed due to the overall structure of the sensor. When one direction is subjected to load, it will inevitably cause deformation in other directions, leading to unexpected response values from the strain gauges.
- Error coupling originates from uncertain factors such as manufacturing processes, bonding methods, and differences in strain gauge performance, and it exhibits distinct individual characteristics.

Through theoretical design and finite element analysis results, this paper demonstrates that the three sets of bridge outputs of the designed strain-based three-dimensional force sensor possess a certain degree of force decoupling. Due to the influence of inter-dimensional coupling issues, the strain outputs in the force sensor are not mutually independent. Therefore, the variation of voltage signal output from the bridge can be represented by the relationship between the elastic body and the applied force:

(17) $$\left[\begin{array}{c} \mathrm{Fx}\\ \mathrm{Fy}\\ \mathrm{Fz} \end{array}\right]=K\left[\begin{array}{c} {\mathrm{Fx}}_{i}\\ {\mathrm{Fy}}_{i}\\ {\mathrm{Fz}}_{i} \end{array}\right]=\left[\begin{array}{ccc} \mathrm{Kxx} & \mathrm{Kxy} & \mathrm{Kxz}\\ \mathrm{Kyx} & \mathrm{Kyy} & \mathrm{Kyz}\\ \mathrm{Kzx} & \mathrm{Kzy} & \mathrm{Kzz} \end{array}\right]*\left[\begin{array}{c} {\mathrm{Fx}}_{i}\\ {\mathrm{Fy}}_{i}\\ {\mathrm{Fz}}_{i} \end{array}\right]$$

In this equation, Fx, Fy, and Fz represent the output values of the pressure loads detected by a three-dimensional force sensor based on the acquisition instrument; K is the coupling matrix; and the physical meaning of Kxy is the output variation caused by a Y-directional force load in the X-direction. Fx_i_, Fy_i_, and Fz_i_ represent the input loads in the three directions.

Multiple sets of known force loads are sequentially applied in the X-, Y-, and Z-directions, and the strain outputs in the three directions corresponding to each force load in these directions are obtained. Then, these data are fitted as straight lines using a linear function. By calculating the slope of each straight line, the corresponding elements in the coupling matrix can be obtained.

The output voltage signal values are recorded when different sets of force loads are applied in different directions. To improve the accuracy of the voltage output signal values, the least squares method is used for decoupling fitting. By performing linear fitting on the output curves, the slope of the output curve of the sensor can be obtained, and subsequently, the coupling errors in each direction of the strain-based three-dimensional force sensor can be calculated.

**Figure 17:**
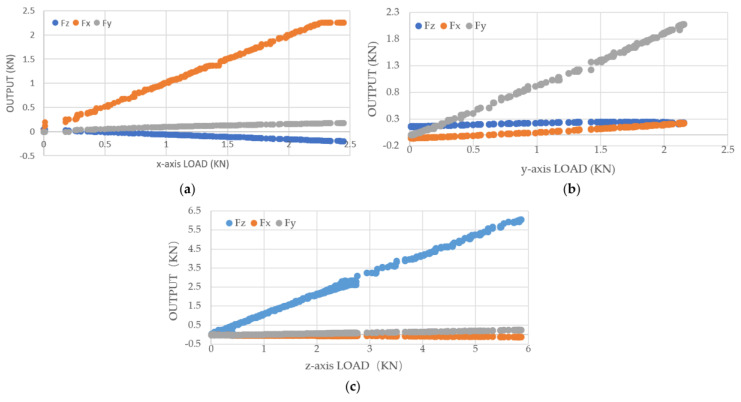
Cross-coupling effect curve: (**a**) *x*-axis direction; (**b**) *y*-axis direction; (**c**) *z*-axis direction.

According to Figure 17, the sensor exhibits high linearity in the three directions, and the inter-axis coupling among the directions is relatively small. By calculating the slope of the fitted line in Figure 17, the following results can be obtained:

(18) $$K=\left[\begin{array}{ccc} 0.9493 & 0.0491 & -0.0203\\ 0.0411 & 0.9692 & 0.0425\\ -0.0448 & 0.0502 & 0.9893 \end{array}\right]$$

Based on Equation (17), it can be inferred that the variation of the three-dimensional signal can be derived when force loads occur in the known elastic body of the sensor in the three directions. Similarly, with the measured signal variations, the magnitude of the three-dimensional force can be inversely deduced. Multiplying both sides of Equation (17) by the inverse matrix of the coupling matrix K yields:

(19) $$\left[\begin{array}{c} {\mathrm{Fx}}_{i}\\ {\mathrm{Fy}}_{i}\\ {\mathrm{Fz}}_{i} \end{array}\right]=\left[\begin{array}{ccc} 1.0569 & -0.0548 & 0.0240\\ -0.0470 & 1.0365 & -0.0455\\ 0.0502 & -0.0551 & 1.0142 \end{array}\right]*\left[\begin{array}{c} \mathrm{Fx}\\ \mathrm{Fy}\\ \mathrm{Fz} \end{array}\right]$$

### 4.3. Performance Test of Force Sensor in Cuting Operation

**Figure 18:**
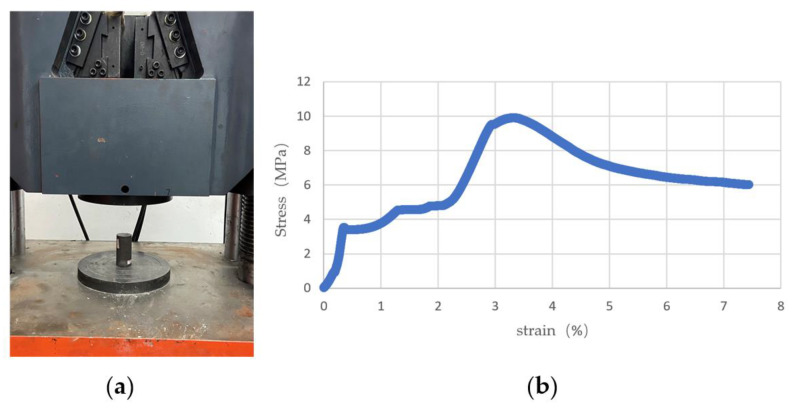
(**a**) The compression testing machine; (**b**) stress-strain curves.

In order to build the artificial coal mass model experimentally, the uniaxial compressive strength (UCS) of artificial coal samples was measured, as shown in Figure 18. The properties of the artificial coal mass are a UCS of 10.05 MPa, Young’s modulus of 0.31 GPa, Poisson’s ratio of 0.32, and BTS of 1.15 MPa.

**Figure 19:**
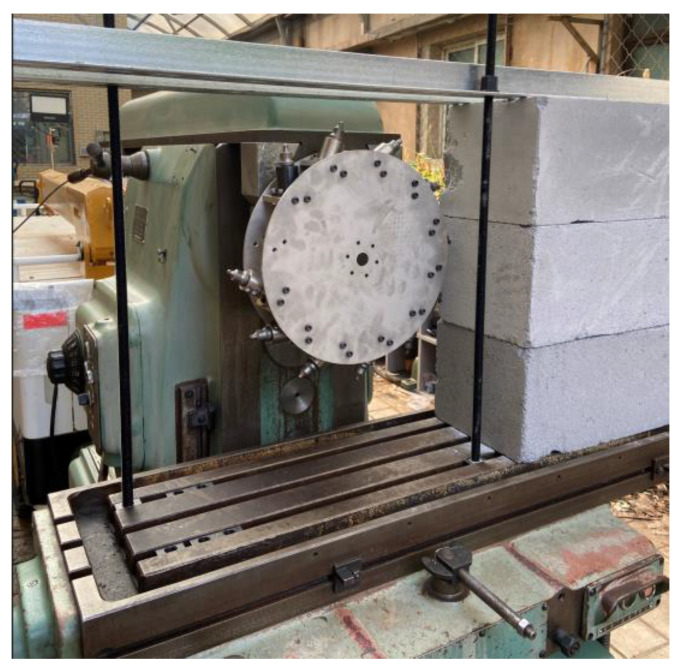
Shearer simulation drum-cutting experimental monitoring device.

We installed the sensors on the simulated drum of the shearer to conduct actual cutting tests, and evaluated and verified the three-dimensional force sensors. The shearer’s simulated drum, equipped with a toothed three-dimensional force sensor, is installed on the spindle of the test bed, which is modified from an XA6132 horizontal milling machine. Then, we installed the cutting sample on the T-shaped workbench and fixed it with angle steel and bolts, as shown in Figure 19.

**Figure 20:**
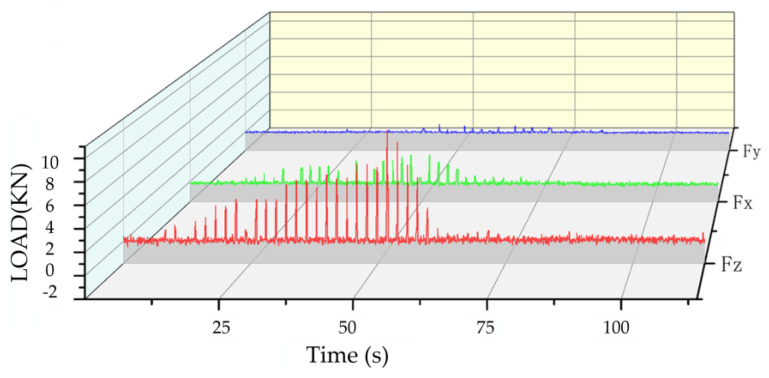
Three-dimensional force sensor cutting force.

We set the shearer drum speed to 30 r/min and the table’s advancing speed to 150 mm/min. The three-dimensional force sensor data are collected, as shown in Figure 20.

The experimental results demonstrate that the newly developed sensor is able to accurately measure the force during the pick cutting operation.

## 5. Conclusions

This paper designs and fabricates a three-dimensional force sensor for measuring the cutting force of a simulated drum pick on a shearer. The sensor adopts 16 high-sensitivity strain gauges and utilizes a Wheatstone bridge circuit in the X-, Y-, and Z-directions for measurement. To enhance the sensitivity of the sensor, the position of the strain gauges is determined through finite element simulation analysis. The dynamic simulation evaluation shows that the sensor has a first-order natural frequency of approximately 1.575 kHz, which meets the requirements for operational conditions. Practical loading tests were carried out using a calibration system for the tooth’s three-dimensional force sensor, and the results demonstrate good linearity, repeatability, and hysteresis. The sensor effectively measures cutting forces in the three directions of the simulated drum pick on the shearer, with sensitivities of 0.748 mv/v, 2.367 mv/v, and 2.83 mv/v, respectively. The cross-sensitivity error is less than 5.02%. The sensitivity of a multi-axis force sensor is typically determined based on its design and manufacturing specifications, rather than a universal standard. Sensitivity is generally related to factors such as the sensor’s measurement range, resolution, and application. The purpose of this experiment was to differentiate between coal and rock based on the magnitude of force (typically with a hardness difference of one-fold). Within the linear range of the sensor, it is desirable to have higher sensitivity because only in high-sensitivity situations will the output signal value corresponding to the measured change be relatively large, which is advantageous for signal processing. However, it should be noted that with higher sensitivity, it is also easier for the sensor to be affected by external noise unrelated to the measurement, and these noises will be amplified by the amplification system, thus affecting the measurement accuracy. In this study, a 24-bit AD converter was used, and with a sensitivity of 0.748 mv/v, the resolution was 0.45‰ for a corresponding range of 5 KN, which was approximately 2.2 N. Cross-sensitivity error can be calculated by solving the relevant influence values using calibration matrices. Therefore, both sensitivity and cross-sensitivity error met the requirements of the experiment. These findings confirm that the sensor’s structure meets the measurement requirements for tooth-cutting forces.

## Figures and Tables

**Table 1:** Parameters of simulated drum.

Name	Value	Name	Value
Drum Diameter	450 mm	Helix Angle	0.2967°
Drum Width	200 mm	Pick Cone Angle:	40°
End Plate Width:	34 mm	Pick Installation Angle	40°
Number of Blade Heads	3	Speed	60 r/min
Number of Blade Sections	4	Traction Speed:	300 mm/min

**Table 2:** Parameters of coal rock specimens.

Name	Value	Name	Value
Tensile Strength:	5.19 MPa	Uniaxial Compression Strength:	12.25 MPa
Elastic Modulus:	0.319 GPa	Poisson’s Ratio	0.331

**Table 3:** Parameters of sensor material.

Materials	Modulus of Elasticity	Poisson’s Ratio	Density
316 stainless steel	2.11 × 10^5^ Mpa	0.3	7980 kg/m^3^

**Table 4:** Parameters of Modal.

Mode	1	2	4	4	5
Freq	1.575 KHz	1.711 KHz	1.712 KHz	3.23 KHz	3.306 KHz

**Table 5:** Parameters of standard pressure sensor and pressure collector.

Parameter Name	Value	Parameter Name	Value
Rated range	500 Kg	Repeatability	0.03%F.S.
Output sensitivity	2.0 ± 10% mV/V	Creep (30 min)	0.03%F.S.
Zero output	0.03% F.S.	Temperature Sensitivity Drift	0.03%F.S.
Non-linearity	0.03% F.S.	Zero Temperature Drift	0.05%F.S.
Hysteresis	0.03% F.S.	Response Frequency	1 KHz

**Table 6:** Parameters of sensor data collector.

Parameter Name	Value	Parameter Name	Value
Comprehensive accuracy	1‰F.S.	Input range	±15 mV
Non-linearity	0.2‰F.S.	Conversion mode	24 bit/Σ-σ
Input impedance	>10 MΩ	Temperature gain drift	20 ppm/°C
Zero adjustment range	±10 mV	Sampling rate	3.2 kps

**Table 7:** Initial measurement parameters of sensor.

Direction	Bridge Supply	Bridge Initial Offset	Full Load Output	Initial Sensitivity
Z	4.7980 V	1.217 mV	4.944 mv/5 KN	0.756 mv/v
X	4.6228 V	0.367 mV	12.137 mv/2 KN	2.494 mv/v
Y	4.7194 V	−0.362 mV	13.693 mv/2 KN	2.920 mv/v

**Table 8:** Sensitivity parameters of sensor.

Direction	Slope	Intercept	Pearson’s r	R^2^
Z	0.9893	0.9892	0.9998	0.9996
X	0.9493	3.7716	0.9980	0.9960
Y	0.9692	−2.64	0.9993	0.9993

**Table 9:** Parameters of sensor.

Cross Direction	Slope	Intercept	Pearson’s r	R^2^
Kxy	0.0491	−9.017	0.9974	0.9949
Kxz	−0.0203	−0.669	−0.9776	0.9557
Kyx	0.0411	0.902	0.9848	0.9700
Kyz	0.0425	−1.923	0.9885	0.9772
Kzx	−0.0448	6.785	−0.9995	0.9990
Kzy	0.0502	10.384	0.9664	0.9340

## Data Availability

The data presented in this study are available upon request from the corresponding author.
